# Cariprazine and clozapine combination for the treatment of psychosis in a young, female patient with schizophrenia: a case report

**DOI:** 10.3389/fpsyt.2024.1452980

**Published:** 2024-08-21

**Authors:** Anzejs Dmuhovskis, Maris Taube

**Affiliations:** ^1^ Faculty of Residency, Riga Stradiņš University, Riga, Latvia; ^2^ Department for Depression and Crisis, Riga Center of Psychiatry and Narcology, Riga, Latvia; ^3^ Department of Psychosomatic Medicine and Psychotherapy, Riga Stradiņš University, Riga, Latvia

**Keywords:** cariprazine, clozapine, psychosis, polypharmacy, treatment, young female

## Abstract

The task of a psychiatrist is to select the most appropriate medication or combination of drugs to treat the symptoms of schizophrenia while minimizing the risk of side effects and ensuring the patient achieves the highest level of functioning possible. This is a challenging task as the action of each drug or group of drugs is different. The efficacy of cariprazine, which affects D3 receptors as a D3/D2 receptor partial agonist, has been extensively studied and is one of the first medication choices by practicing psychiatrists when treating patients with negative symptomatology. In this clinical case, we demonstrate the effective and safe treatment of a patient’s positive and affective symptoms using a combination of cariprazine, clozapine, and venlafaxine.

## Introduction

Schizophrenia affects approximately 1% of the population (1) and is characterized by positive symptoms such as hallucinations (voices), delusions, abnormalities in thought processes, thought withdrawal, insertion, broadcasting, and the belief that feelings and actions are being controlled by external forces. Negative symptoms of schizophrenia include poverty of speech, affective flattening, lack of motivation, lack of pleasure (anhedonia), loss of drive, and a diminished ability to express feelings. In addition, there are cognitive deficits (attention, memory, language impairment) and affective symptoms (depression) that may also be present (2).

It is important to treat the different symptoms of schizophrenia, and various medications are used to target specific groups of symptoms. There is more experience (and greater effectiveness) in treating positive symptoms, with negative and cognitive symptoms of schizophrenia being more difficult to manage (3). Cariprazine has demonstrated good treatment effectiveness, particularly for negative symptoms due to its effect on D3 receptors as a D3/D2 receptor partial agonist (4). However, in clinical practice, the choice of medication is determined by the patient’s previous, and often negative, treatment experience, the insufficient effectiveness of drugs and other treatments tried, the experienced and expected side effects of the medication, the severity of the symptoms, and the patient’s current level of functioning and desired level of functionality in the future (e.g., the desire to work). Treatment with multiple medications is not well-supported in treatment guidelines, thus, the choice of medications or combinations of medications is a difficult task for the psychiatrist. However, polypharmacy is a common treatment practice and has yielded useful combinations (5). The clozapine and cariprazine combination may be one useful treatment choice for some difficult cases.

This case report would be valuable and will narrow the gender gap, because women are still underrepresented in clinical trials investigating medications in psychiatry (6).

## Case presentation

A 20-year-old female patient was first treated in a psychiatric hospital in 2020 (at the age of 17) in an acute psychotic state due to pronounced suicidal thoughts and suicidal behavior. The patient’s decision to commit suicide was preceded by an acute psychotic state with commenting and commanding auditory hallucinations. The presence of a first-rank symptom—hearing voices that gave a continuous commentary—resulted in a diagnosis of schizophrenia in accordance with the International Classification of Diseases 10th revision classification system (7). The intervention of the patient’s friend helped her get medical care. At the psychiatric hospital, the patient’s condition improved, and her psychotic symptoms were reduced. She initially received the antipsychotic drug risperidone. However, her prolactin blood levels increased, and risperidone was switched to quetiapine. The patient was discharged from the hospital after 20 days of treatment with directions to continue with outpatient treatment with a recommended dose of 200 mg of quetiapine per day. Following hospital discharge, the patient did not feel well, was unable to function fully, had little energy or motivation, and her mood was low. The patient then visited various psychiatrists searching for a better solution. Antipsychotic medications were also switched during this time, with aripiprazole up to 15 mg and olanzapine up to 10 mg tried, but with insufficient effect. The patient’s condition then worsened; she developed severe anxiety, the commenting voices began, and she had intrusive thoughts. The patient was unable to work, even though she had found a job, and was repeatedly admitted to a psychiatric hospital in 2023 where her treatment included cariprazine, clozapine, as well as the extended-release antidepressant, venlafaxine. The patient’s condition significantly improved, and she has returned to work with no health complaints.

### Background history

The patient’s mother suffers from depression and has received treatment in a mental health hospital specializing in depression. The patient comes from a family of five children. After finishing school, she studied art. However, she does not work in the arts and has found temporary employment in trade. There is no history of somatic health problems, she has a supportive family, and her mother is understanding of her health problems.

### Treatment episodes

The patient was first hospitalized in a psychiatric ward in September 2020, due to having expressed suicidal thoughts and writing a suicide note. The patient’s behavior was determined by auditory hallucinations. Specifically, a female’s voice in the patient’s head was criticizing and threatening her. The patient also believed foreign thoughts were being inserted into her mind. The voices commanded her to commit suicide, and the patient was uncommunicative and crying. Risperidone inpatient treatment was initiated and titrated up to a dosage of 4 mg per day. The voices then became quieter, and the patient felt better. Unfortunately, risperidone raised her prolactin level to 3697.5 mIU/L (8, 9). After withdrawing the risperidone, her prolactin levels returned to normal. After 20 days of treatment in the patient was discharged from the hospital. The use of quetiapine 200 mg/day was recommended for regular treatment and prevention of psychosis, and to avoid negative side effects (10).

The second admission to a psychiatric hospital took place in July of 2023. The patient heard voices expressing reproaches and threats. She also had severe anxiety, a significantly lowered mood, was not eating well, was nauseous, had lost weight, and was crying. At this moment, depressive symptoms were an important part of the presentation of disease during an episode of psychosis.

Inpatient treatment with cariprazine was started, reaching a dose of 6 mg/day with a concurrent administration of clozapine, gradually increased to 100 mg/day. Venlafaxine XR (extended-release) was also simultaneously started, with the dosage increased to 75 mg/day. **Table 1** lists the inpatient psychiatric treatments the patient received, which included an impression of her mental status following an examination by a psychiatrist and the results of assessments using the Clinical Global Impression - Severity scale (CGI-S) and the CGI-improvement (CGI-I) scale.

**Table 1:** Timeline of patient events, medications, scoring (CGI-S, CGI-I), and mental status description (7/07/2023–4/08/2023).

Date	Event/ treatment day	CGI-S	CGI-I	Medication/s	Mental Status description
07/07/2023	Hospitalisation	7		Cariprazine (CAR) 3 mg/day/p/o, Clozapine (CLO) 37.5 mg/day/p/o, Venlafaxine (VEN) extended-release (XR) 75 mg/day/ p/o, music, art, drama, dance therapy, occupational therapy, psychotherapy, physiotherapy	Hears accusing voices in her head, low mood, anxiety, unable to concentrate and work. Insufficient understanding of the disease. Has suicidal thoughts because voices are telling her to do it. Difficult to communicate with people. Hospitalization required.
11/07/2023	5^th^ treatment day	6	3	Increase dosage of clozapine (CLO) to 50 mg/day/p/o	
12/07/2023	6^th^ treatment day	6	3	Increase dosage of clozapine (CLO) to 75 mg/day/p/o	
13/07/2023	7^th^ treatment day	5	3	Increase dosage of cariprazine (CAR) to 6 mg/day/p/o	
14/07/2023	8^th^ treatment day	5	3	Increase dosage of clozapine to 100 mg/day/p/o	
4/08/2023	Discharge from mental hospital	2	1	Cariprazine (CAR) 6 mg/day/p/o, Clozapine (CLO) 100 mg/day/p/o, Venlafaxine (VEN) extended-release (XR) 100 mg/day/ p/o	No psychotic symptoms, mood has improved, no anxiety, more energy, positive thoughts about the future, wants to work and plans to return to work. Easier to communicate with others, keeps the conversation going.

At the hospital, the patient also received non-drug therapy (e.g., visual art, music, drama, dance, and movement therapy, physiotherapy, psychotherapy, and occupational therapy) (11). After a 28-day course of treatment, the patient was no longer hearing voices, was able to concentrate, and her mood had significantly improved. She smiled, maintained conversations, showed interest in life, and had strength, energy, and a better appetite. Furthermore, the patient wished to return to work.

### Current status

Following her hospital discharge, the patient has continued to regularly take a maintenance therapy dose of prolonged-release venlafaxine at 75 mg/day, 100 mg of clozapine, and 6 mg of cariprazine. The patient feels well and is employed. Currently, her health status has nearly completely stabilized, and she has no subjective complaints and no side effects from medications.

### Patient perspective

After a hospital stay, the patient has returned to work. She is feeling well and has a positive outlook on the future. The patient has no complaints or medication side effects and regularly visits a psychiatrist on an outpatient basis.

## Discussion

The patient’s disease manifestations included a combination of the different domains of schizophrenia symptoms. Acute and severe psychotic disorders with commanding and suicidal auditory hallucinations are a characteristic manifestation of positive symptoms, while the lowering of mood belongs to the domain of affective symptoms.

While the patient’s treatment with risperidone was effective during her first hospitalization, it was complicated by an increase in prolactin levels. Given this development, the treatment plan was changed from risperidone to quetiapine. Because quetiapine also will cause a hyperprolactinemia (12), decision were made to use the lowest possible dose. This decision was made while considering the patient’s young age, the risk of sexual dysfunction, and the potential for future pregnancy.

When treating the patient’s recurrent psychosis, the choice of therapy was based on the following considerations: the expression of positive symptoms with the risk of suicide, the expressiveness of affective symptoms and anxiety, the risk of increased prolactin levels, and the need to restore and preserve her functionality, including cognitive abilities. The combination of cariprazine and clozapine (5, 13, 14) was an opportunity to balance different therapeutic goals and effectively treat the psychotic symptoms of schizophrenia. Clinical guidelines recommend that suicidal patients consider clozapine earlier even if they are not treatment-resistant (15). Clozapine monotherapy could be considered for this treatment episode. The clinician’s decision in favor of Clozapine and Cariprazine combination was made based on the clinical situation – high risk of suicide if monotherapy fails, and necessary to address negative symptoms and improve social functioning in the long term (14). In that clinical situation, the psychiatrist had limited options for other (third) unsuccessful treatment choices.

Clozapine is usually, but not exclusively, used for treatment-resistant schizophrenia (16, 17). In our case, the patient had pronounced suicidal ideations (18), and several other antipsychotic medications were not effective. Thus, the therapeutic efficacy, the effect on suicidal thoughts (19), and the insufficient effectiveness of other antipsychotic medications led to the choice of clozapine.

Cariprazine was used to prevent possible negative symptoms and reduce cognitive symptoms. This helped to promote patient cooperation in the use of medications and ensure the best possible functioning after inpatient treatment.

Venlafaxine helped correct affective (depressive) symptoms, reduce anxiety, and increase the patient’s energy and activity levels (20). The guidelines recommend first optimizing the antipsychotic dose (12), but in this case, dose optimization was challenging due to the increasing risk of elevated prolactin levels.

Our patient had no significant side effects from the medications started during her second hospitalization. No orthostatic hypotension from clozapine or akathisia from the cariprazine was observed. This allowed for the rapid escalation of medication doses that quickly reduced psychotic symptoms, which is important for patients with acute psychosis.

### Limitations

There are several limitations concerning the approach taken in this case. First, no other clinical scales were used for patient assessment other than the CGI-S and the CGI-I. Another aspect of the patient’s treatment that could have affected her stabilization was the non-drug therapy (visual art, music, drama, dance and movement therapy, physiotherapy, psychotherapy, and occupational therapy) she was provided. The role of venlafaxine is also ambiguous, as cariprazine also has an anti-depressive effect. Thus, its necessity in adding it to the treatment scheme is unclear.

## Conclusion

Prescribing the medication combination of cariprazine and clozapine may be a suitable treatment approach, especially for young women with early-onset schizophrenia. This is particularly true if the patient has a heightened risk of increased prolactin production and suicidal ideation. This approach also ensures the treatment of different symptom domains (positive, negative, affective, and cognitive) and recovery.

## Data Availability

The original contributions presented in the study are included in the article/supplementary material. Further inquiries can be directed to the corresponding author.
